# Bioinformatics services for analyzing massive genomic datasets

**DOI:** 10.5808/GI.2020.18.1.e8

**Published:** 2020-03-31

**Authors:** Gunhwan Ko, Pan-Gyu Kim, Youngbum Cho, Seongmun Jeong, Jae-Yoon Kim, Kyoung Hyoun Kim, Ho-Yeon Lee, Jiyeon Han, Namhee Yu, Seokjin Ham, Insoon Jang, Byunghee Kang, Sunguk Shin, Lian Kim, Seung-Won Lee, Dougu Nam, Jihyun F. Kim, Namshin Kim, Seon-Young Kim, Sanghyuk Lee, Tae-Young Roh, Byungwook Lee

**Affiliations:** 1 Korea Bioinformation Center (KOBIC), KRIBB, Daejeon 34141, Korea; 2 Genome Editing Research Center, KRIBB, Daejeon 34141, Korea; 3 Department of BioInformation Science, Ewha Womans University, Seoul 03760, Korea; 4 Department of Life Sciences and Division of Integrative Biosciences & Biotechnology, Pohang University of Science & Technology (POSTECH), Pohang 37673, Korea; 5 Department of Systems, Biology Division of Life Sciences, and Institute for Life Science and Biotechnology, Yonsei University, Seoul 03722, Korea; 6 Bioposh Inc., Daejeon 34016, Korea; 7 SeqGenesis, Daejeon 34016, Korea; 8 School of Life Sciences, Ulsan National Institute of Science and Technology, Ulsan 44919, Korea; 9 Strategic Initiative for Microbiomes in Agriculture and Food, Yonsei University, Seoul 03722, Korea; 10 Genome Structure Research Center, KRIBB, Daejeon 34141, Korea; 11 SysGenLab Inc., Pohang 37613, Korea

**Keywords:** analysis pipeline, cloud computing, genomic data, web server, workflow system

## Abstract

The explosive growth of next-generation sequencing data has resulted in ultra-large-scale datasets and ensuing computational problems. In Korea, the amount of genomic data has been increasing rapidly in the recent years. Leveraging these big data requires researchers to use large-scale computational resources and analysis pipelines. A promising solution for addressing this computational challenge is cloud computing, where CPUs, memory, storage, and programs are accessible in the form of virtual machines. Here, we present a cloud computing-based system, Bio-Express, that provides user-friendly, cost-effective analysis of massive genomic datasets. Bio-Express is loaded with predefined multi-omics data analysis pipelines, which are divided into genome, transcriptome, epigenome, and metagenome pipelines. Users can employ predefined pipelines or create a new pipeline for analyzing their own omics data. We also developed several web-based services for facilitating downstream analysis of genome data. Bio-Express web service is freely available at https://www.bioexpress.re.kr/.

## Introduction

Next-generation sequencing (NGS) technology has revolutionized the researches in biology and medicine during the last decade. It is routinely used in genomics field, and explosive growth of NGS data has resulted in ultra-large-scale datasets and various computational problems [1]. Public archives for sequencing data such as the Sequence Read Archive have grown rapidly and now exhibit a doubling time of 10–18 months [2]. In Korea, genomic data have been increasing rapidly in recent years. As of February 2020, approximately 277 TB of genomic data have been deposited in Korea Bioinformation Center (KOBIC) database.

It is not easy for typical researchers to analyze these massive genomic datasets. To obtain results from the data, researchers need to use high-performance computing (HPC) environments with sufficient storage space and CPU cores. In addition, the difficulties in creating complicated computational pipelines and maintaining software packages tend to overwhelm bench biologists and prevent them from attempting to analyze their own genomic data [3]. Despite the availability of a vast set of computational tools and methods for genomic data analysis in public, it is still challenging for a genomic researcher to organize these tools, integrate them into workable pipelines, find accessible computational platforms, configure the computing environment, and perform the actual analysis.

A promising solution to address this computational challenge is cloud computing, where CPUs, memory, and storage are accessible in the form of virtual machines [4]. The cloud computing, by definition, refers to the on-demand delivery of IT resources and applications via the Internet [5]. The Software as a Service (SaaS) cloud service for applications provides the perfect solution for the analysis of massive genomic datasets. SaaS is a method of software delivery in the IT field that allows data to be accessed from any device with an Internet connection and web browser. In recent years, cloud computing has rapidly emerged as a viable option for quickly and easily acquiring computational resources and pipelines for large-scale NGS data analyses [6].

The parallelism techniques in HPC infrastructure are used to process all the produced data in a feasible time [7]. Parallel computing is a type of computation in which many calculations or the execution of processes are carried out simultaneously. However, it is still challenging to integrate bioinformatics experiments with parallel techniques in the HPC environments. Many applications developed for the analysis of genomic data are either tools running only on a parallel platform, such as a MapReduce platform, or general-purpose (mainly Linux-based) programs. It is crucial to integrate these two types of platform-based applications on a single pipeline.

In this study, we present Bio-Express, a software package for deploying an on-demand computing cloud with minimal user intervention. The goal of Bio-Express is to provide a web-based analysis environment in which all genomic researchers, including those with limited or no programming knowledge, can easily analyze their own genomic data. The Bio-Express Graphic User Interface (GUI) provides a workflow editor in which users can simply use a predefined analysis pipeline or create a multistep analysis pipeline using preinstalled programs. The analysis pipelines on Bio-Express are exactly reproducible, and all analysis parameters and inputs are permanently recorded. Bio-Express makes it simple to perform a multistep analysis using simple drag and drop functionality. We also developed several web-based services for facilitating downstream analysis of genome data such as gene-set enrichment analysis.

## Methods

### Hardware

All runs of analysis pipelines on Bio-Express are performed on a cluster of five master nodes and 33 data nodes. The hardware system of Bio-Express consists of 800 core CPUs, 2 TB of memory, and 800 TB of disk storage in total. Each node has an Intel Xeon E502690 v2 3.0 GHz CPU, 96 GB of memory, and 28 TB of disk storage. The data node HDD configuration consists of the Hadoop Distributed File System and a solid-state drive (SSD) cache. The node manager handles the individual data nodes in a Hadoop cluster.

### Graphic User Interface (GUI)

**Fig. 1.**
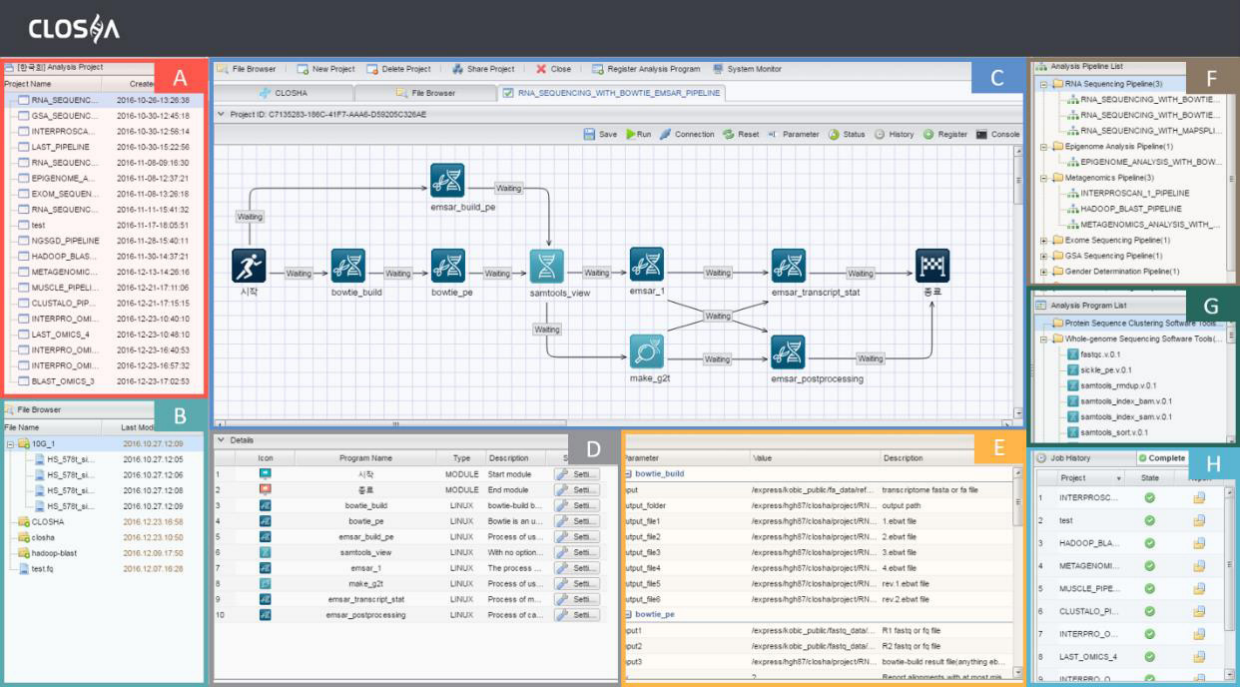
The interface of the Bio-Express workspace. The Bio-Express workflow editor has eight panels: the user’s projects (A), the file explorer (B), the canvas (C), the analysis programs of the current pipeline (D), the program parameter settings (E), the pipeline list (F), the program list (G), and the job execution history (H).

The GUI workspace of Bio-Express consists of eight panels: the user’s projects, the file explorer, the canvas, the analysis programs of the current pipeline, the program parameter settings, the pipeline panel, the program panel, and the job execution history (Fig. 1). Among these panels, the canvas is the most important panel and is used for creating and modifying workflows by arranging and connecting activities to drive processes. The canvas provides the working surface for creating new workflows or editing predefined ones. The canvas makes it simple to perform multistep analyses using drag and drop functionality.

### Pipelines

The workflows, or analysis pipelines, in the canvas are commonly depicted as directed acyclical graphs, in which each of the vertices has a unique identifier and represents a task to be performed. Additionally, each of the tasks in a workflow can receive inputs and produce outputs. The outputs of a task can be directed through another task as an input. An edge between two vertices represents the channeling of an output from one task into another. The edges determine the logical sequence. A task can be executed once all of its inputs can be resolved. If one of user pipeline programs fails, users can select the program of the pipeline to view more detailed information on errors, and resume the whole pipeline from the failed program after fixing the errors.

### The transfer of data

The bottleneck of cloud computing is the transfer of data into clouds. Therefore, we developed a fast file transfer tool, Gbox, for uploading massive genomic datasets to the cloud server from the user’s local computer and for downloading the resulting files to the local. The client program of Gbox can be downloaded from the website and be installed on the user’s computer. Gbox has a file transfer at a rate of approximately 10 Gigabits per second, capable of dealing with big data over the web. Currently, Gbox has no file size limitations and storage limit on the Bio-Express cloud server.

### Scalability

Scalability is one of the most attractive prospects of cloud computing and provides a useful safety net when a user’s needs or demands change. The resource and job manager of Bio-Express distributes computing resources to user jobs within a parallel computing infrastructure. Its aim is to satisfy user’s demands for computation and achieve a good performance in overall system’s utilization by efficiently assigning jobs to resources. The resource and job manager analyzes the application performance during runtime and predicts the demand for load balancing, i.e., when to add/remove resources or redistribute workload. Thus the scalability of Bio-Express improves the execution speed of job by efficient assignment of computing resources.

## Results

The analysis pipelines can be divided into two types: predefined and user-created. As of February 2020, Bio-Express contains approximately 170 analysis tools and 57 predefined analysis pipelines for genome, transcriptome, epigenome, and metagenome data. Users can employ a predefined pipeline suitable for their data by selecting a pipeline in the pipeline panel. If users want to create a new analysis pipeline, they can build their own pipeline either from scratch or by modifying a predefined pipeline. The following sections describe representative predefined analysis pipelines in the pipeline panel.

### Genome pipeline

For the analysis of genome data and high-density single nucleotide polymorphism (SNP)-arrays, we developed 25 pipelines and programs, which can be grouped into seven categories: (1) discovery of variants for human, animal and plant data, (2) discovery of candidate genes from whole-exome sequencing data of rare diseases, (3) identification of somatic mutations, SNPs and short INDELs from cancer genomes, (4) clonality and evolutionary analysis of cancer genomes, (5) structural variation and copy-number analysis of whole-genome sequencing data, (6) population genomic analysis of whole-genome sequencing data, and (7) association studies and genomic predictions from the high-density SNP-arrays. We also developed two dockerized workflows that can be used for the discovery of SNPs, short INDELs, or copy-number variations from germline and somatic sample data, and for population genomics analysis in evolutionary studies. The two dockerized workflows were developed using the Workflow Description Language, developed on the Data Sciences Platform at the Broad Institute.

We developed two tools using genome data: GenoCore [8] and SEXCMD [9]. GenoCore is a new method for selecting a core collection using modified statistical measures related to genetic allele coverage and diversity. It can be used to select core subsets from plant genotype datasets, which is important for increasing cost-effectiveness and shortening the time required for the analyses of genome-wide association studies (GWAS), genomics-assisted breeding of crop species, etc. SEXCMD is a pipeline that can extract sex marker sequences from reference sex chromosomes and rapidly identify the sex of individuals from whole-exome/genome and RNA sequencing (RNA-Seq) data.

### Transcriptome pipeline

**Fig. 2.**
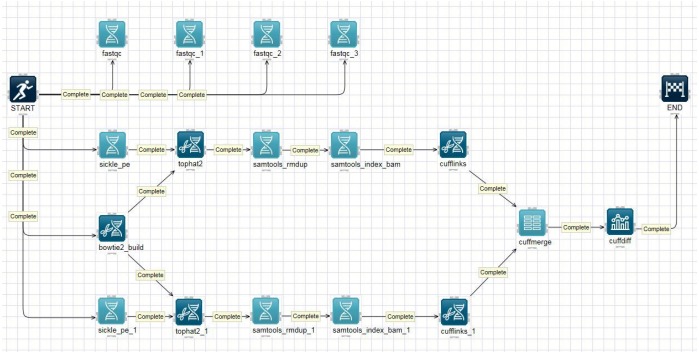
Screenshot of the RNA-sequencing (RNA-Seq) schematic diagram and its pipeline. The RNA-Seq pipeline was implemented on the canvas.

The analyzing an organism’s transcriptome is important for understanding the functional elements of a genome [10]. RNA-Seq is a deep-sequencing technique that can be used to explore and profile the entire transcriptome of any organism [11]. Fig. 2 shows a typical schematic overview of the RNA-Seq analysis pipeline on the canvas. The pipeline, often referred to as the tuxedo pipeline, includes five analysis tools: TopHat 2.1.1 [12], Cufflinks 2.1.1 [13], Cuffmerge 2.1.1, Cuffdiff 2.1.1, and limma voom 1.0 [14]. TopHat is a fast splice junction mapper that is used to align RNA-Seq reads to large genomes and analyze the mapping results to identify splicing junctions between exons. Cufflinks is used to assemble these alignments into a parsimonious set of transcripts and then estimate the relative abundances of these transcripts. The main purpose of Cuffmerge is to merge several Cufflinks assemblies, making it easier to produce an assembly GTF file suitable for use with Cuffdiff. Cuffdiff is then used to identify significant changes in transcript expression, splicing, and promoter use. Finally, voom robustly estimates the mean-variance relationship and generates a precision weight for each individual normalized observation, which can be used to calculate differentially expressed genes from transcript expression levels. Several other pipelines for RNA-Seq data analysis are available at Bio-Express, including MapSplice2-RSEM [15], Bowtie-EMSAR [16], STAR-HTSeq [17], and STAR-RSEM [18],

### Epigenome pipeline

**Fig. 3.**
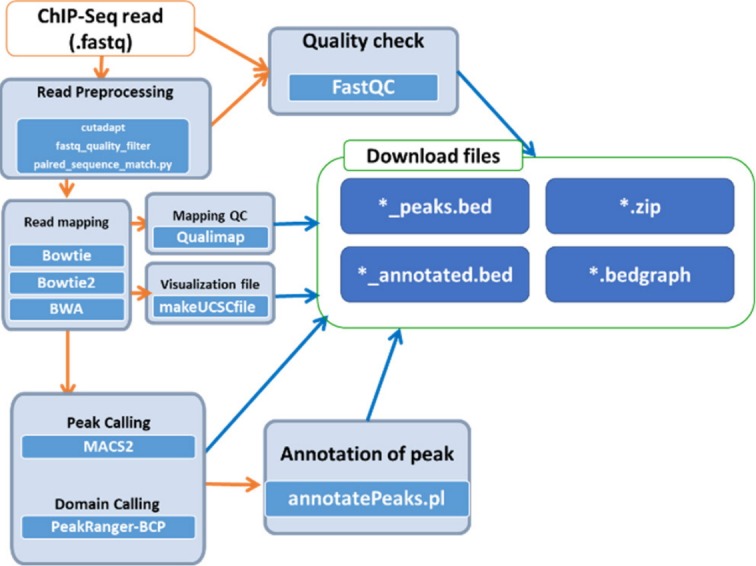
Workflow for the histone modification analysis pipeline. ChIP-Seq, chromatin immunoprecipitation sequencing.

Epigenetic changes, including histone modifications and DNA methylation, provide a differential gene regulatory mechanism without altering DNA sequences [19]. Histone modifications occur mostly at histone tails by acetylation, methylation, phosphorylation, and ubiquitination. The accurate mapping of the called peaks of these modification sites is a critical step for understanding epigenetic transcriptional regulation. A popular, fast applicable pipeline for histone modification mapping was established by comparing various peak calling programs such as CisGenome [20], MACS1 and MACS2 [21], PeakSeq [22] and SISSRs [23], RSEG [24], SICER [25], hiddenDomains [26], BroadPeak [27], PeakRanger-CCAT, and PeakRanger-BCP [28]. For the best performance to define the exact binding sites of proteins in DNA, we tested 12 histone modifications using different peak calling programs, and we suggest the MAC2 program for narrow peak identification and PeakRanger-BCP for broad peak identification. The analysis pipeline for histone modifications is summarized in Fig. 3; the input files in fastq format are preprocessed by cudapt, fastq_quality_filter, and paired_sequence_match.py and then read quality is tested with FastQC. After mapping reads onto the reference genome, peak calling or domain calling is followed by application of MACS2 or PeakRanger-BCP. The final output is produced with annotation information. This simple pipeline is open to the public under the Bio-Express portal provided by KOBIC.

### Metagenome pipelines

**Fig. 4.**
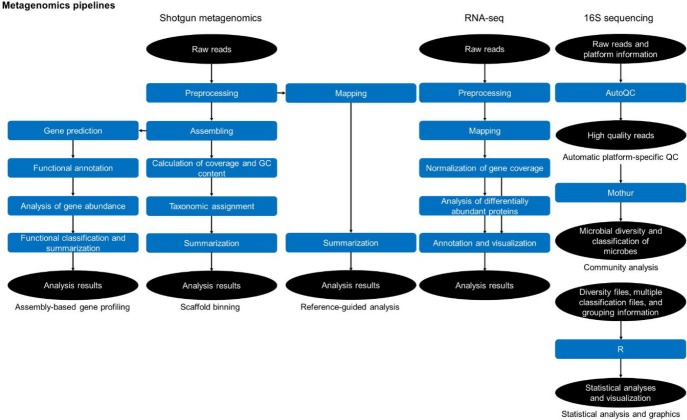
Simplified workflow diagram of the metagenomics pipelines.

The analysis of metagenome data can be categorized into three parts (Fig. 4): whole metagenome shotgun sequence data analysis (shotgun metagenomics), whole transcriptome shotgun sequence data analysis (RNA-Seq), and 16S rRNA gene amplicon sequence data analysis (16S sequencing). In shotgun metagenomics, there are three pipelines: the assembly-based gene profiling, scaffold-binning, and reference-guided analysis pipelines. In the assembly-based gene profiling pipeline, sequence reads are assembled using SOAPdenovo-63mer [29]; gene regions in the assembled sequences are predicted using MetaGeneMark [18], and the functions of the gene regions are assigned by the BLAST program with the COG and GenBank nr databases.

In the scaffold-binning pipeline, the coverage and GC content of the scaffolds are calculated, and taxonomic identifiers are assigned to the scaffolds using MEGAN [30] and HMMER 3.0 [31]. In the reference-guided analysis pipeline, sequence reads are mapped with the BWA program with reference genes or genomes. In the RNA-Seq category, sequence reads are mapped and normalized, statistical analyses are performed to identify differentially abundant genes, and finally, the results are annotated.

The 16S sequencing category is composed of three modules in sequential order: automatic platform-specific quality control (QC), community analysis, and statistical analysis and graphics. We developed a program, AutoQC, for the automatic platform-specific QC module. AutoQC uses platform-specific conditions to efficiently remove erroneous reads. AutoQC is freely available at https://sourceforge.net/projects/autoqc/. The community analysis module mainly reveals the microbial diversity and classification of microbes using Mothur. In the statistical analysis and graphical statistical analyses like pMANOVA test [32] are performed and the analysis results are visualized.

### Creating custom (user defined) pipelines

Users can create a new pipeline to analyze their own data on the canvas. To create a new pipeline, users click the ‘New Pipeline’ button in the top menu and select an analysis pipeline type. Users will have only the [Start] and [End] modules on the canvas immediately upon creating a pipeline after selecting a ‘new analysis pipeline design’ in the project type. Users can drag and drop their desired analysis programs from the list of analysis programs on the right of the canvas. After the positioning of a desired analysis program on the canvas, when the users place the mouse over the edge of the analysis program icon, a connection mark will be created that can be drawn to the module. Starting from the mark, the connector must be dragged until the icon of the next analysis program to be connected becomes translucent. Users can make connections to the start module, the analysis program and the end module using this method to perform the analysis. The path for the output file is automatically a sub-path of the project in setting the input data. Finally, the analysis pipeline is executed with a message that the analysis has started. The status of the project is displayed on a real-time basis in three modes: Complete, Execute, and Wait.

**Fig. 5.**
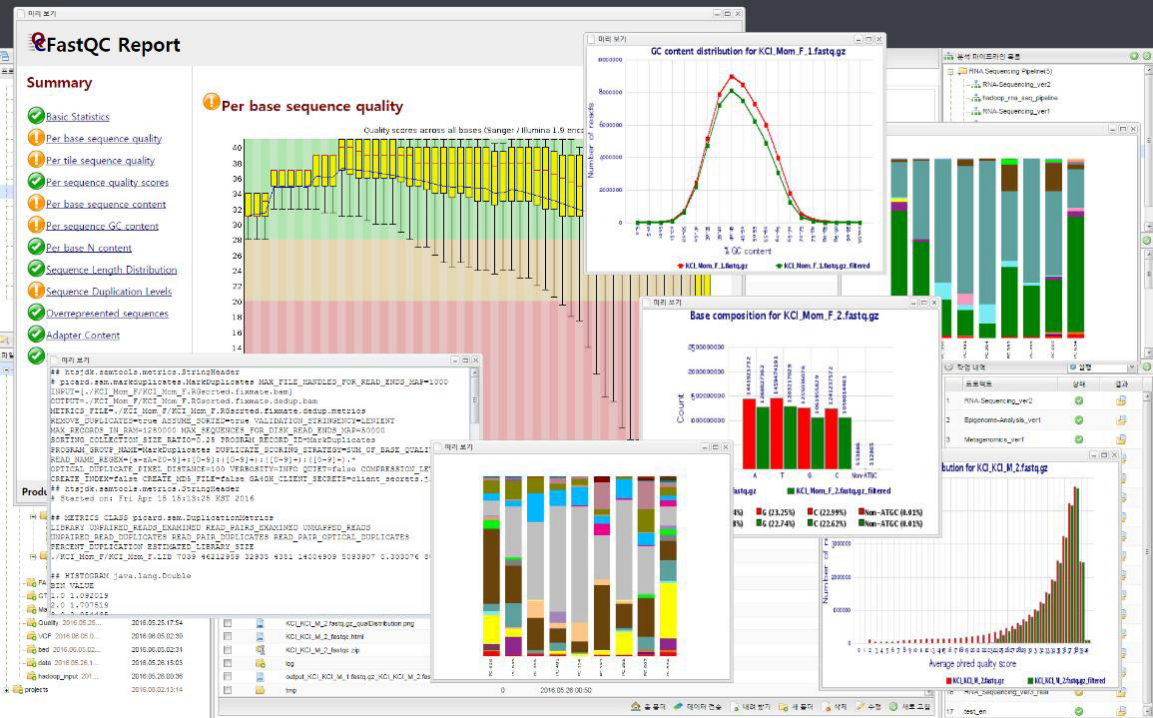
Screenshot of Bio-Express results. Users can view files in various formats, including text, HTML, and PNG on the web.

Users can see the final results by clicking the ‘Results’ icon on the menu and downloading them to the user’s local computer by clicking the ‘Download’ button on the menu bar. Bio-Express also allows users to view files in various formats including text, HTML, and PNG on the screen without having to download the files (Fig. 5).

### Web servers

We have developed traditional web servers in which the input is a small amount of data such as a gene list. The traditional web servers do not provide automatic scalability to the applications which is the major feature of the cloud server [33]. The developed web servers are ADGO2 [34], ExPathNet [35], GSA-SNP [36], and Barcas [37].

ADGO2 provides biological interpretations of microarray data (gene-set enrichment approach) and a list of genes (gene list overrepresentation approach) via composite annotation. ADGO2 also supports gene- or sample-permuting gene-set enrichment analysis for RNA-Seq count data. ExPathNet provides network-weighted gene-set clustering that incorporates both gene-set overlap and protein-protein interaction networks. GSA-SNP is standalone software that provides widely used GSA methods for SNP and GWAS data. GSA-SNP2 [38] is an improved version of GSA-SNP that provides fast high-power computation by incorporating the random set model and SNP-count adjusted gene scores. GSA-SNP2 can also visualize protein interaction networks within and across the significant pathways. Barcas is pharmacogenomics data analysis software developed for the mapping and analysis of multiplexed barcode sequencing data. Barcas employs a trie data structure for fast mapping with mismatches allowed and provides many functions, including quality control, data analysis and visualization. Table 1 shows the web servers used for gene-set, pathway, and pharmacogenomic data analysis.

### Comparison between Bio-Express and Galaxy

We compared Bio-Express with Galaxy, an open source system that is the most widely used pipeline system and empowers non-computational users to do computational biology. We performed a comparison experiment between Bio-Express and Galaxy with the same data and the same RNA-Seq pipeline. We used an RNA-Seq case-control sample data set: 42,112,235 paired-end case reads and 40,975,645 paired-end control reads. The total sample size of the case and the control reads is approximately 42 GB. We assigned four CPU cores and 16 GB of memory for a single RNA-Seq job. The same machine was used for the comparison. The execution of the RNA-Seq pipeline on the sample data using Bio-Express takes a total of 3 h 44 min. The execution time using Galaxy was 6 h 11 min, showing Bio-Express has approximately 1.7 times better performance than Galaxy in the execution of the RNA-Seq pipeline. There are two main reasons for the difference in runtime between the two systems. As Galaxy internally processes intermediate data for data conversion after finishing each pipeline program, the execution time is slightly increased due to the internal process of each step. Secondly, Bio-Express has fast access to input and output data by fully utilizing the function of a SSD cache, compared to the Galaxy system.

## Discussion

The substantial decrease in the cost of NGS techniques in the past decade has dramatically reshaped the genome research and has led to its rapid adoption in biological research. Nowadays, massive amount of data can be generated quickly using NGS platforms. These data range from the function and regulation of genes, the clinical diagnosis and treatment of diseases, to the omics profiling of individual patients for precision medicine. With the exponential increase in volume and complexity of NGS data, cluster or HPC systems are essential for the analysis of large amounts of NGS data. But the associated costs with the infrastructure itself and the maintenance personnel will likely be prohibitive for small institutions or laboratories.

Cloud-based applications and resources have been developed specifically to address the computational challenges of working with very large volumes of data generated by NGS technology. Cloud computing has changed how we manage computational resources. Increasingly cloud computing is also changing how large computational resources are organized and how scientists in genomics collaborate and deal with vast genome data sets.

We presented a Hadoop based distributed computational framework for large-scale genomic analysis, called Bio-Express, which incorporates a variety of tools and methods. Our system offers a variety of services to researchers. Firstly, Bio-Express allows genomic researchers without informatics or programming expertise to perform complex large-scale analysis with only a web browser using drag and drop functionality. Secondly, Bio-Express is a hybrid system that enables users to use both analysis programs providing traditional tools and MapReduce-based big data analysis programs simultaneously in a single pipeline. Lastly, we also developed a high-speed data transmission solution, Gbox, to transmit a large amount of data at a fast rate.

In the future work, we continuing to add powerful pipelines and programs including the most popular sequence and genome analysis algorithms, and to enable accessible and reproducible genomic science. Secondly, we plan to create a framework with both client-side and server-side components that simplifies the development of web-based visual applications. Visualization and visual analysis are important tools in high-throughput genomics experiments because large datasets do not need to be downloaded. Lastly, we will create a standalone installation package of Bio-Express. The increasingly large size of many datasets and moving the huge datasets is one particularly challenging aspect of current and future genomic science. Hence, local Bio-Express installations near the data are likely to become more prevalent because it makes more sense to run Bio-Express locally as compared to moving the data to a remote Bio-Express server.

## Figures and Tables

**Table 1.** Web servers for gene-set, pathway, and pharmacogenomic data analysis

Tool	Main function	Web address	
ADGO2	Gene-set analysis of microarray data	http://www.btool.org/ADGO2	
ExPathNet	Gene-set analysis with network-weighted clustering	http://epn.appex.kr/epn/	
GSA-SNP2	Gene-set analysis of GWAS summary data	https://sites.google.com/view/gsasnp2	
Barcas	Software for analyzing barcode-seq data	http://medical-genome.kribb.re.kr/barseq/	

GWAS, genome-wide association studies.
